# Investigating research study participant compensation practices at a California academic and research institution

**DOI:** 10.1017/cts.2025.57

**Published:** 2025-03-31

**Authors:** Mariam Carson, Paula Fleisher, Rana Barar, Li Zhang, Elizabeth Tioupine, Hilary Seligman

**Affiliations:** 1 School of Medicine, University of California San Francisco, San Francisco, CA, USA; 2 Clinical and Translational Science Institute, University of California San Francisco, San Francisco, CA, USA; 3 Department of Medicine, University of California San Francisco, San Francisco, CA, USA; 4 Department of Epidemiology and Biostatistics, University of California San Francisco, San Francisco, CA, USA; 5 Institutional Review Board, University of California San Francisco, San Francisco, CA, USA; 6 Department of Family and Community Medicine, University of California San Francisco, San Francisco, CA, USA

**Keywords:** Research participant, research compensation, research reimbursement, IRB, institutional review board, research, study participant, ethics

## Abstract

**Introduction:**

While providing compensation for participation in research studies is common, there is an ongoing debate surrounding compensation models and how they can be equitably applied. This work attempts to better understand the landscape of research compensation by evaluating factors associated with compensation of research study participants across instiutional review board (IRB)-approved studies at a single academic institution in California.

**Methods:**

We extracted all IRB applications for social, behavioral, educational, and public policy research studies between January 1, 2019, and December 31, 2021, at the University of California, San Francisco. Compensation amounts, time estimates for participation, and location of study activities (hybrid, remote, in-person) were extracted from free text entries in the IRB application and reorganized into discrete variables. Multivariable logistic regression was used to assess factors associated with receiving payment after adjusting for time.

**Results:**

We analyzed 403 unique IRB applications. Studies held at public hospitals and clinics were more likely to provide compensation to study participants, whereas studies held at the university hospitals and clinics were less likely to provide compensation. Unfunded studies also were less likely to provide compensation to research study participants. While participants that were classified as “economically/educationally disadvantaged” and “unable to read, speak, or understand English” within the institution’s IRB application were more likely to receive compensation, those that had “diminished capacity to consent” were less likely to receive compensation.

**Conclusions:**

While there are multiple frameworks for compensation, there is still significant variability in compensation strategies. Institutions should center equity in considering standardized approaches to compensation for research participation.

## Introduction

Compensating people for participation in research studies is an expected and common, though not universal, practice. The ethics of how, why, and how much to compensate for research participation has been richly debated, yielding a general lack of consensus surrounding optimal compensation models [1–5]. Pandya and Desai outline four “traditional” compensation models that consider participants’ backgrounds and experiences [1]. Ultimately, it is up to the investigator to weigh the benefits and costs of each compensation model and select the model that best fits their research needs and target population, and it is up to institutional review boards (IRBs) to endorse the chosen model. Given the historically exploitative nature of research, investigators and their institutions must consider equity when designing research studies and compensation practices [6]. Increased compensation for research study participation has been shown to increase the diversity of diverse study cohorts, which improves the overall quality and validity of research [6,7].

One common ethical concern among all compensation strategies is coercion; however, recommendations for avoiding coercion in participant study compensation are also widely debated. Anderson proposes multiple strategies that can be tailored to compensation, including payments based on local living wages, guaranteed compensation for participation-related injuries, and a national tracking system to ensure participants are not enrolling in multiple concurrent studies [8]. Gelinas et al. propose reimbursement for any out-of-pocket expenditures by participants, compensation for any burden to the participant (including time), and offering payments as incentives for recruitment and retention only [9]. While there is consensus that research study participants should be compensated, equitable compensation strategies continue to be debated, and the burden of determining what is “equitable” typically lies with the investigator and the ethical advisory boards that approve their research.

In addition to various compensation models, there are different approaches among researchers for compensation amounts within similar fields of study [2,4]. Although several studies have evaluated IRB applications to assess participant compensation models across multiple institutions, little is known about the variability of compensation amounts or compensation modalities (ranging from cash to physical gift cards to electronic payment apps) within a single academic institution. Furthermore, while many research participation remuneration models incorporate ethical frameworks, to our knowledge, none of them address the disparities and inequities that arise from structural racism and oppression. Compensation that is effective and just for an individual with consistent income, benefits, and privilege may not be so for an individual who has experienced trauma due to historical and contemporary racism. It is unclear whether and how remuneration strategies address the complex dynamic between individuals from communities that experience oppression and medical and research institutions. For example, some investigators offer higher remuneration to “difficult-to-reach” populations, which may be an effective strategy partially because it addresses the additional burden to research participation for populations historically excluded from or unethically involved in research.

We sought to better understand these gaps by evaluating the range of compensation values and modalities available to researchers, as well as the compensation values and modalities provided to participants, across IRB-approved studies at a single academic institution, with the goal of better understanding what studies, what people, and what circumstances are associated with higher compensation values and different compensation platforms. We hope that this work will allow us to better understand research practices taking place within the institution, illuminate places where racism may be “showing up” in institutional research practices, and inform guidelines for more equitable compensation strategies grounded in anti-racism and anti-oppression.

## Methods

We extracted all IRB applications for social, behavioral, educational, and public policy research studies between January 1, 2019, and December 31, 2021, at the University of California, San Francisco (UCSF). The UCSF IRB represents several locations throughout the Bay Area, including university hospitals and clinics, public hospitals and clinics, and Veterans Affairs (VA) hospitals and clinics. A detailed list of inclusion/exclusion criteria for the IRB applications included in this study can be found in Appendix 1.

We focused our analysis on social, behavioral, educational, and public policy research studies, including interviews, focus groups, or surveys. Focusing on non-invasive, low-risk research allowed us to limit our scope to compensation primarily for participants’ time and travel to and from study activities. Thus, it is valid to compare across studies. Comparing compensation for studies involving activities such as biospecimen extraction or surgical procedures introduces additional ethical complexities that should be examined in future studies.

Studies that contained more than one study group and compensation schedule were disaggregated into unique records, with unique study groups and compensation for each group forming record. For example, if a single study included interviews with patients compensating $100 as well as focus groups with healthcare providers compensating $200, that single study produced two records. Compensation amounts, time estimates for participation, and location of study activities (hybrid, remote, in-person) were extracted from free text entries in the IRB application and reorganized into discrete variables. All data analysis was performed at the record level.

Frequencies and percentages were used to describe categorical data. Multivariable logistic regression was used to assess factors associated with receiving payment after adjusting for time as a covariate. Several variables were not mutually exclusive (e.g., study sites and study populations), and therefore were included and analyzed in the logistic regression model separately. Statistical significance was declared at *p* < 0.05. No multiple comparisons adjustment was used. Statistical software R (R version 4.0.5) was used for analysis.

Appendix 1 further outlines data cleaning procedures and analysis.

## Results

We reviewed 403 unique IRB applications. Some applications involved multiple distinct study participant groups (for example, providers and patients), which were disaggregated into 574 unique records consisting of a distinct study participant group and an associated compensation value. Records were excluded due to missing payment information (*n* = 30), missing participant time requirements (n = 28), and time requirements exceeding 10,000 min (*n* = 5), resulting in a total of 359 IRB applications and 511 records included in the data analysis. A summary of the IRB applications included in the analysis is provided in Table 1.

**Table 1.** Summary of IRB application demographics

Variable	Total N (%)	No payment (%)	Any payment (%)
Total IRB applications	359 (100)	119 (33)	240 (67)
Study site*			
University hospitals and clinics	296 (82.5)	109 (91.6)	187 (77.9)
Public hospitals and clinics	80 (22.3)	13 (10.9)	67 (27.9)
VA hospitals and clinics	8 (2.2)	0 (0)	8 (3.3)
Department of public health	23 (6.4)	2 (1.7)	21 (8.8)
Other	2 (0.6)	0 (0)	2 (0.8)
Year			
2019	104 (29)	32 (26.9)	72 (30)
2020	142 (39.6)	55 (46.2)	87 (36.2)
2021	113 (31.5)	32 (26.9)	81 (33.8)
Funding source			
Federal/other government	113 (31.5)	18 (15.1)	95 (39.6)
Other funding	74 (20.6)	15 (12.6)	59 (24.6)
Unfunded	172 (47.9)	86 (72.3)	86 (35.8)

* Because study sites are not mutually exclusive, sum of sites is greater than 359.

Table 2 demonstrates the odds of receiving any payment for participation in a research study by study site, adjusted for time required for study participation. Participating in a study at a public hospital/clinic site was associated with three times higher odds of receiving payment (OR 3.06, 95% CI [1.79–5.49], *p* < 0.001) when compared to all other study sites, whereas participating in a study at a university hospital or clinic resulted in 60% lower odds of receiving payment (*p* = 0.004) when compared to all other study sites.

**Table 2.** Payment odds by study site, adjusted for time required for study participation (multivariable)

	OR*	95% CI, *p*-value
University hospitals and clinics	0.40	0.21-0.73, *p* = 0.004
Public hospitals and clinics	3.06	1.79-5.49, *p* < 0.001
VA hospitals and clinics	1.11	0.36-4.15, *p* = 0.869
Department of public health	5.45	1.88-23.21, *p* = 0.006

* The odds ratio compares each study site to a referent group that includes all other study sites.

Table 3 displays different sources of funding for studies, adjusted for time required for study participation. Compared to federal funding and government funding, participants in unfunded studies had 66% lower odds of receiving payment (*p* < 0.001). Table 3 also displays different categories of participants that qualify as “vulnerable populations” that receive extra protection during research. Studies that specifically recruited participants who were economically and/or educationally disadvantaged had almost four times greater odds of providing payment compared to those who did not (OR 3.92, 95% CI [2.33–6.88], *p* < 0.001). Studies planning to enroll non-English speaking participants were also more likely to provide payment for study participation (OR 2.76, 95% CI [1.64–4.83], *p* < 0.001). Studies recruiting participants with diminished capacity to consent were less likely to receive payment (OR 0.33, 95% CI [0.13–0.83], *p* = 0.017) when compared to studies recruiting all other types of special study populations.

**Table 3.** Compensation by funding source and study population, adjusted for time required (multivariable)

		OR*	95% CI, *p*-value
Funding type	Federal/other government	Ref	–
	Other funding	1.15	0.62-2.18, *p* = 0.653
	Unfunded	0.34	0.21-0.55, *p* < 0.001
Study population	Economically/educationally disadvantaged	3.92	2.33-6.88, *p* < 0.001
	Subjects unable to read, speak, or understand English	2.76	1.64-4.83, *p* < 0.001
	Diminished capacity to consent	0.33	0.13-0.83, *p* = 0.017
	Prisoners	0.79	0.08-17.22, *p* = 0.852
	Age<18	1.03	0.64-1.69, *p* = 0.902

* The referent group for odds ratios in the funding type rows is federal or other government funding. All other study populations are the referent group for odds ratios in the study population rows. For example, the referent for economically/educationally disadvantaged studies is all studies that were not identified as economically/educationally disadvantaged, and the referent group for age <18 is all studies that included age >18.

## Discussion

This study examined the receipt of compensation across multiple research studies within a single academic institution. Our results show that research studies held at public hospitals and clinics were more likely to compensate study participants, whereas studies held at the university hospitals and clinics were less likely to provide compensation. Unfunded studies also were less likely to provide compensation to research study participants. While participants who were classified as “economically/educationally disadvantaged” and “unable to read, speak or understand English” within the institution’s IRB application were more likely to receive compensation, those that had “diminished capacity to consent” were less likely to receive compensation.

This study, showing significant variation in compensation odds for similar studies in a single institution, is consistent with other evaluations of compensation for research study participation. For example, in one investigation evaluating 467 research studies approved by 11 different IRB, compensation values for participation in studies ranging from short-term studies to long-term clinical trials ranged from $5 to $2000 [2]. However, there were no attempts to explain or justify this variation by adjusting for time required or other study risks [2]. Another study evaluated IRB-approved protocols from 69 principal investigators within three multicenter pediatric clinical trials, revealing similar variation in participant compensation amount and rationale [4]. Participants were reimbursed for travel/parking/food by 33 PIs, for time by 13 PIs, and for “inconvenience” by 22 PIs [4]. The authors highlight the variability of compensation amounts among researchers, even within the same site or study.

There have been efforts to develop and update ethical frameworks for equitable research study participant compensation [8–11]. Millum and Garnett discuss how participant compensation can be coercive in the form of subjection [12]. Studies risk coercion by subjection when participants feel obligated to enroll in a study to avoid an unacceptable outcome (for example, persistence of low socioeconomic status) [12]. This is especially salient when researchers and participants have different motivations for study enrollment (for example, researchers are motivated by scientific discovery, whereas participants are motivated by financial incentives) [12]. By providing compensation, researchers ensure that enrollment in the study will help the participant avoid—or at least mitigate—the unacceptable outcome. Research has shown that many IRBs view any payment as coercive and are especially averse to high levels of payment [7,13]. Furthermore, studies that pose greater than minimal risk to participants face additional ethical challenges surrounding compensation and coercion compared to studies that pose minimal risk. Bierer et al. argue that equitable compensation and coercion are two related but distinct ethical issues [6]. Insufficient payment or no payment at all can impact study recruitment and retention which, in addition to being unjust, also has ethical implications when study populations are not representative of the true population demographic.[6] Walter et al. demonstrate that increased payment is associated with the proportional demographic representation of racial/ethnic groups [7]. As investigators and their IRBs consider compensation for research study participation, equitable compensation strategies should be considered separately from coercion.

There are several limitations to this study. The current structure of the IRB application is insufficient for a thorough health equity analysis; the data likely undercount vulnerable populations, as the list of “vulnerable populations” available from which investigators must choose within the IRB application is limited and vague. Furthermore, some studies may list certain vulnerable populations as part of their participant recruitment pool but ultimately may not directly recruit or enroll that group. Details of study remuneration strategies were also inconsistent, as study summaries were free text entries with somewhat limited clarity on exact participant compensation values and time required for participation. Study locations also refer to where the investigator team is housed, not the locations from where research participants were recruited. Finally, our analysis did not include raffles (where study participants were entered into a lottery system to win a single or limited number of compensatory prizes). We also recognize that there may be other potential confounders in this observational study that may be unaccounted for, as well as potential interaction effects between multiple records nested within a single IRB application. These results are observational and do not establish a causal relationship between research study population, site, funding source, and compensation amount. Finally, we recognize that our sample only included low-risk social, behavioral, educational, and public policy research studies, which is only a small subset of research that requires human participation. Future research should investigate compensation strategies for more invasive and high-risk studies.

However, we believe these results can help inform recommendations for IRB guidelines and institutional best practices for providing compensation to research study participants. Such recommendations include more structured data fields instead of free text responses so that IRBs can more consistently monitor equitable research practices. Institutions can also require investigators to provide compensation amounts and time required for research study participants in structured entry fields within the application.

Compensating research study participants for their time and contributions to the scientific process is an important component of research and essential to assuring equitable access to research participation. While there are multiple frameworks for compensation, there is still significant variability in compensation strategies among investigators within a single institution. Institutions should center equity in considering how to approach compensation for research participation. In order to develop an equity-centered framework for research study participant compensation, institutions must commit to collecting information within their IRB applications that hold investigators accountable to providing clear and accurate compensation practices. These data can then be used to develop best practices for investigators when designing future research studies.

## Supporting information

Carson et al. supplementary material Carson et al. supplementary material

## Supplementary material

The supplementary material for this article can be found at https://doi.org/10.1017/cts.2025.57.

## References

[1] Pandya M , Desai C. Compensation in clinical research: the debate continues. Perspect Clin Res. 2013;4(1):70. doi: 10.4103/2229-3485.106394. 23533986 PMC3601710

[2] Grady C , Dickert N , Jawetz T , Gensler G , Emanuel E. An analysis of U.S. practices of paying research participants. Contemp Clin Trials. 2005;26(3):365–375. doi: 10.1016/J.CCT.2005.02.003. 15911470

[3] Reiser SJ. Research compensation and the monetarization of medicine. JAMA. 2005;293(5):613–614. doi: 10.1001/JAMA.293.5.613. 15687317

[4] Kimberly MB , Hoehn KS , Feudtner C , Nelson RM , Schreiner M. Variation in standards of research compensation and child assent practices: a comparison of 69 Institutional review board-approved informed permission and assent forms for 3 Multicenter pediatric clinical trials. Pediatrics. 2006;117(5):1706–1711. doi: 10.1542/PEDS.2005-1233. 16651328

[5] Mduluza T , Midzi N , Duruza D , Ndebele P. Study participants incentives, compensation and reimbursement in resource-constrained settings. Bmc Med Ethics. 2013;14(S1):1-1–11. doi: 10.1186/1472-6939-14-S1-S4. 24564948 PMC3878327

[6] Bierer BE , White SA , Gelinas L , Strauss DH. Fair payment and just benefits to enhance diversity in clinical research. J Clin Transl Sci. 2021;5(1):159–160. doi: 10.1017/CTS.2021.816. PMC8427546 34527298

[7] Walter JK , Burke JF , Davis MM. Research participation by low-income and racial/Ethnic minority groups: how payment may change the balance. Clin Transl Sci. 2013;6(5):363–371. doi: 10.1111/CTS.12084. 24127923 PMC5350891

[8] Anderson EE. A proposal for fair compensation for research participants. AJOB. 2019;19(9):62–64. doi: 10.1080/15265161.2019.1630501. 31543033

[9] Gelinas L , Largent EA , Cohen IG , Kornetsky S , Bierer BE , Fernandez Lynch H. A framework for ethical payment to research participants. N Engl J Med. 2018;378(8):766–771. doi: 10.1056/NEJMSB1710591. 29466147

[10] Dominguez D , Jawara M , Martino N , Sinaii N , Grady C. Commonly performed procedures in clinical research: a benchmark for payment. Contemp Clin Trials. 2012;33(5):860–868. doi: 10.1016/J.CCT.2012.05.001. 22580210 PMC3408804

[11] Swanson DM , Betensky RA. Research participant compensation: a matter of statistical inference as well as ethics. Contemp Clin Trials. 2015;45:265–269. doi: 10.1016/J.CCT.2015.08.014. 26334678 PMC4674311

[12] Millum J , Garnett M. How payment for research participation can be coercive. AJOB. 2019;19(9):21–31. doi: 10.1080/15265161.2019.1630497. 31419191

[13] Largent EA , Grady C , Miller FC , Wertheimer A. Money, coercion, and undue inducement: aa survey of attitudes about payments to research participants. IRB. 2012;34(1):1. Accessed December 5, 2023. /pmc/articles/PMC4214066/. PMC4214066 22338401

