# Supplementary material for: Investigating research study participant compensation practices at a California academic and research institution
Source: J Clin Transl Sci. 2025 Mar 31;9(1):e103. doi: 10.1017/cts.2025.57 (PMC12089853; doi:10.1017/cts.2025.57)
Supplement: Carson et al. supplementary material [file S2059866125000573sup001.docx]

**Appendix**

**Table A:** Inclusion/Exclusion criteria for IRB applications

| **Inclusion Criteria** | **Exclusion Criteria** |
| --- | --- |
| Expedited review applications | Study activities taking place outside of the US |
| IRB approved between 1/1/2019 and 12/31/2021 | Clinical, basic, and biomedical research studies |
| Social, behavioral, educational, and public policy studies | Studies with biospecimen extraction |
|  | IRB exempt studies |

**Table B:** Variables extracted from IRB applications

| **Variable** | **Definition** |
| --- | --- |
| Subjects Paid? | Are research participants compensated? (Y/N) |
| Cash | Research participants compensated with cash |
| Check | Research participants compensated with check (Y/N) |
| Gift Card | Research participants compensated with gift card (Y/N) |
| Other | Research participants compensated with item not listed (Y/N) |
| Subjects not paid | Research participants not compensated (Y/N) |
| Debit Card | Research participants compensated with debit card (Y/N) |
| Reimbursement | Research participants reimbursed for travel and parking expenses only (Y/N) |
| UCSF Card | Research participants compensated with UCSF-specific card (Y/N) |
| Payment Amount | Amount of compensation |
| Additional Incentives | Free text for PIs to detail non-cash compensation modalities |
| Coronavirus | Research study related to COVID-19 (Y/N) |
| Funding | Funding source for research study |
| Location | Location of study activities (in-person, remote, hybrid) |
| Time Required | Time commitment for research study participant (including time for travel, consent) |
| 0-6 years | Research participant aged 0-6 (Y/N) |
| 7-12 years | Research participant aged 7-12 (Y/N) |
| 13-17 years | Research participant aged 13-17 (Y/N) |
| 18-64 years | Research participant aged 18-64 (Y/N) |
| 65+ years | Research participant aged 65+ (Y/N) |
| Unable to consent | Research participants include participants unable to provide consent (Y/N) |
| Diminished capacity to consent | Research participants include participants with diminished capacity to provide consent (Y/N) |
| Subjects unable to read, speak, or understand English | Research participants include participants unable to read, speak, or understand English (Y/N) |
| Prisoners | Research participants include prisoners (Y/N) |
| Economically/educationally disadvantaged | Research participants include economically and/or educationally disadvantaged participants (Y/N) |
